# Extracellular vesicles in triple-negative breast cancer: current updates, challenges and future prospects

**DOI:** 10.3389/fmolb.2025.1561464

**Published:** 2025-04-14

**Authors:** Prashant Kumar Tiwari, Anis Ahmad Chaudhary, Saurabh Gupta, Mandeep Chouhan, Himanshu Narayan Singh, Sarvesh Rustagi, Salah-Ud-Din Khan, Sanjay Kumar

**Affiliations:** ^1^ Biological and Bio-Computational Lab, Department of Life Sciences, School of Basic Sciences and Research, Sharda University, Greater Noida, Uttar Pradesh, India; ^2^ Department of Biology, College of Science, Imam Mohammad Ibn Saud Islamic University (IMSIU), Riyadh, Saudi Arabia; ^3^ Department of Biotechnology, GLA University, Mathura, Uttar Pradesh, India; ^4^ Department of Systems Biology, Columbia University Irving Medical Center, New York, NY, United States; ^5^ Department of Food Technology, School of Applied and Life science, Uttaranchal University, Dehradun, Uttarakhand, India; ^6^ Department of Biochemistry, College of Medicine, Imam Mohammad Ibn Saud Islamic University (IMSIU), Riyadh, Saudi Arabia

**Keywords:** extracellular vesicles, biomarkers, therapeutic challenges, non-coding RNAs, drug delivery

## Abstract

Breast cancer (BC) remains a complex and widespread problem, affecting millions of women worldwide, Among the various subtypes of BC, triple-negative breast cancer (TNBC) is particularly challenging, representing approximately 20% of all BC cases, and the survival rate of TNBC patients is generally worse than other subtypes of BC. TNBC is a heterogeneous disease characterized by lack of expression of three receptors: estrogen (ER), progesterone (PR), and human epidermal growth factor receptor 2 (HER2), resulting conventional hormonal therapies are ineffective for its management. Despite various therapeutic approaches have been explored, but no definitive solution has been found yet for TNBC. Current treatments options are chemotherapy, immunotherapy, radiotherapy and surgery, although, these therapies have some limitations, such as the development of resistance to anti-cancer drugs, and off-target toxicity, which remain primary obstacles and significant challenges for TNBC. Several findings have shown that EVs exhibit significant therapeutic promise in many diseases, and a similar important role has been observed in various types of tumor. Studies suggest that EVs may offer a potential solution for the management of TNBC. This review highlights the multifaceted roles of EVs in TNBC, emphasizing their involvement in disease progression, diagnosis and therapeutic approach, as well as their potential as biomarkers and drug delivery.

## 1 Introduction

Cancer remains a major concern worldwide, after skin cancer, BC is the most common among women. According to NIH statistical data, about 3 lakh cases were expected with 7.1% mortality rate. BC increasing steadily over the past two decades (Aravindan et al., 2024), and one of the most prevalent diseases affecting women worldwide (Arnold et al., 2022). Several studies and their results from different perspectives unanimously classify BC as a highly heterogeneous disease molecular and histological both level (Viale, 2012). Over time, several molecular markers have been discovered to classify BC based on factors like genomic instability, genetic changes, and gene activity. Advanced technologies have made it much easier for us to understand why BC is so diverse by identifying biomarkers like ER, PR, and HER2. These markers have helped classify BC into five subtypes, including luminal A, luminal B, HER2-enriched, triple-negative (or basal-like), and normal-like breast cancer. This classification helps to predict disease progression and choose effective treatments (Zubair et al., 2021). According to cancer statistics and several studies, the proportion of TNBC is higher in Asian countries. Primarily, Indian data shows that 100,000 people are diagnosed with breast cancer every year. It is estimated that by 2025, global cancer cases will reach approximately 30 million, while deaths will increase to 17 million. Like the United States, breast cancer is the second leading cause of cancer-related deaths in India after lung cancer. The diagnosis of breast cancer presents a significant challenge in effectively managing the disease. According to WHO, survival rates vary across regions, ranging from about 90% in high-income countries, 60% in India and 40% in South Africa (Gupta et al., 2024).

Of all these subtypes of BC, TNBC has received significant attention. TNBC is an aggressive subtype that represent around 11%–20% of all BC cases (Loizides and Constantinidou, 2023). It is characterized by the lack of estrogen receptor (ER), progesterone receptor (PR), as well as lack of overexpression or amplification of human epidermal growth factor receptor 2 (HER2) (Tiwari et al., 2023). Consequently, TNBC is frequently unresponsive to hormone-based therapies, which specifically target ER and PR, as well as strategy designed to address HER2 receptors. The lack of these receptor targets hinders the effectiveness of conventional hormone-based and HER2-targeted therapies in managing TNBC cases (Saleh et al., 2021). TNBC primarily affects young, premenopausal women to a more significant number and has been observed more frequently in African-American women. This is often associated with inherited gene mutations involving the BRCA1 and BRCA2 genes (Howard and Olopade, 2021). The highly aggressive nature of TNBC poses a significant challenge in its diagnosis and prognosis. TNBC have more propensity to metastasize to different body parts in compression of other subtypes of BC (Azim et al., 2020).

Prognostic biomarkers may play in significant role in the initial diagnosis of TNBC. Lipids, circulating tumors DNA (ctDNA), glycogen, tumors-infiltrating lymphocytes (TILs), immune checkpoint molecules (PD-L1), circulating tumors cells (CTCs), and microRNAs (miRNAs) are considered as next-generation predictive biomarkers and promise significant potential for enhance the prognosis of TNBC. Glycogen and lipid show pathology-associated metabolic changes and give important insights into malignancy growth and treatment response. ctDNA serves as a non-invasive methodology to assess tumors genetics and monitor pathological conditions (Alismail, 2024; Banerjee et al., 2024). Patients with early-stage TNBC who have not received adjuvant or neoadjuvant chemotherapy have been shown to have significantly better survival when they have significant quantities of TILs in their BC tissues. These studies results confirm, abundance of TILs in BC may serve as a crucial prognostic factor for early-stage TNBC patients (Leon-Ferre et al., 2024). PD-L1, a protein that involved in immune evasion, mostly exhibit in aggressive type neoplasm. In BC, its expression is related with high histologic grade and negative hormone receptor status. Approximately 20% of TNBC tumors express PD-L1 (Mittendorf et al., 2014).

Currently, the primary therapeutic approach for TNBC involve a combination of surgery, radiation, chemotherapy, and neoadjuvant therapy (Baranova et al., 2022). Though there are some approved chemotherapeutics such as platinum agent (carboplatin and cisplatin), doxorubicin, paclitaxel, capecitabine, gemcitabine, and eribulin, but their efficacy are limited (Twelves et al., 2016). Moreover, most of these drugs cannot cross the blood-brain barrier, posing a challenge in treating brain tumors resulting from TNBC metastasis. Approximately one-third of TNBC patients develop brain metastases, which currently have no available cure, leading to short survival times (Kadamkulam Syriac et al., 2023; Kannan and Cheng, 2023). Some TNBC patients with BRCA1/2 mutations can receive intervention with poly (ADP-ribose) polymerase inhibitors like olaparib and talazoparib, however these options are limited (Hobbs et al., 2021). Therefore, there is a pressing need for developing new and effective therapies with high specificity for BC and minimal damage to healthy tissues.

EVs are tiny particles surrounded by lipid bilayers that are released into the circulation by various types cells, including tumor cells, and these diverse membranous vesicles secreted into the extracellular space (Dixson et al., 2023), which engage in numerous functions, including intercellular communication, immune regulation, disease progression and development, and tissue repair. Additionally, EVs carries cargo such as, proteins, lipids, metabolites DNA and various type RNA, which play a crucial role in biological processes (Yue et al., 2023). Initially, until the late 1990s, EVs did not receive significant attention in the field of research, because in the early stages it was believed that these vesicles were waste material of cells (Yuan et al., 2023). Primarily based on their biogenesis and size, EVs have been classified into three different types (Liu and Wang, 2023), (i) Exosomes range in size from 30 to 150 nm. Their biogenesis occurs within multivesicular bodies (MVBs) via the endocytic pathway, following ESCRT-dependent complexes, and they are released into the extracellular space via exocytosis (Yin et al., 2023). The presence of various biomarkers including tetraspanins (CD9, CD63, CD81), endosomal proteins (TSG101, Rab-GTPase), and heat shock chaperones (HSP70, HSP90) characterize exosomes (Banerjee and Rajeswari, 2023). (ii) Microvesicles are formed through outward of the plasma membrane under stimuli and calcium-dependent pathways. These vesicles range in size from 50 to 1,000 nm and are released through outward budding and fission of the membrane. These include various biomarkers including flotillin-2, CD40 ligand, and annexin (Rani et al., 2023), and (iii) apoptotic bodies which is greater than 1,000 nm in size, formed during the apoptosis process, compared to other EVs it has phosphatidylserine, cytochrome c and DNA histones as major markers (Wen et al., 2023) (Detailed classification and characteristics of EVs mentioned in Table 1).

**TABLE 1:** Extracellular vesicle classification, based on category, size, formation, pathways and marker.

1.	Category	Size	Formation	Biogenesis pathway	Markers	References
2.	Exosomes	30–120 nm	Multivesicular bodies fusion (MVBs) with the plasma membrane	ESCRT-dependent	Tetraspanins (CD9, CD63, CD81), Endosome system proteins (TSG101, Rab-GTPase), and Heat shock chaperones (HSP70, HSP90)	Xu et al. (2022); Banerjee and Rajeswari (2023)
3.	Microvesicle	40–1,000 nm	Outward blebbing of the plasma membrane	Stimuli-dependent, Ca2+-dependent, cell-dependent	Flotllin-2, CD40 ligand, Selectin, Annexin 1	Ståhl et al. (2019)
4.	Apoptotic bodies	>1,000 nm	Plasma membrane budding of Apoptotic cells	Apoptosis-related	Phosphatidylserine, Annexin V, DNA histones	Ståhl et al. (2019), Hu et al. (2020b)

Tumor cell-derived EVs promote tumor development and metastasis through diverse mechanisms by influencing the tumor microenvironment (TME), modulating cellular interactions, and signaling pathways (Tian et al., 2023). TME play crucial role in the TNBC progression, by utilizing different biological mechanisms such as immune suppression, proliferation, angiogenesis, and apoptosis inhibition. Dynamic interactivity between surrounding stromal, endothelial, immune cells, and neoplasm cells builds niche, that facilitated tumor development, metastasis, and epithelial-to-TNBC stem cell transition (Deepak et al., 2020). TME composed diverse types of cells and biological molecule, including ECM components, immune cells, tumor-associated fibroblasts (CAFs), blood vessels and cancer stem cells (CSCs), ECM generate signals for numerus key processes like cell proliferation, replicative immortality, invasion, and apoptosis evasion. CAFs are prime contributors to drug resistance and disease progression by producing growth factors and chemokines, additionally play crucial role in immune cell infiltration (Otranto et al., 2012).

EVs can be collected from various bodily fluids such as blood or urine, providing a non-invasive method to obtain real-time information about the status and types of malignant cells (Kalra et al., 2016). They got special attention in the clinical field due to verity of function like precisely targeted drug delivery (vaccines and therapeutic agents), interaction with specific cell and tissue. This observation has led to the exploration of EVs as potential cargo carriers for delivering (Wang et al., 2020). Notably EVs secreted from body enable them to work well with the same because they are natural and are less likely to cause an adverse reaction or be seen as foreign particles by the immune system of body (Song et al., 2022). EVs surface is made of the cellular proteins and can escape from our immune system. Additionally they can also pass through the protective blood-brain barrier and prevent drugs from breaking down (Kooijmans et al., 2016).

## 2 Biogenesis of extracellular vesicle

Endosomes are membrane-bound compartments within cells that play an essential role in sorting and trafficking various cellular materials, including proteins and lipids. The early endosome is the first step in the endocytic pathway, where the material is internalized from the cell surface via endocytosis (Simonetti et al., 2023). Endosomes mature into late endosomes or multivesicular bodies (MVBs), and specific cargo inside the endosomal membrane forms tiny buds that separate from the membrane. These buds synthesize tiny intraluminal vesicles (ILVs) within the endosome’s inner space, and these ILVs are later released through exocytosis. So based on this process exosomes biogenesis can be categorized into two main pathways, i.e., ESCRT (endosomal sorting complex required for transport)-dependent pathway and the ESCRT-independent pathway (Han et al., 2022).

### 2.1 Biogenesis of exosomes through ESCRT-dependent pathway

**FIGURE 1:**
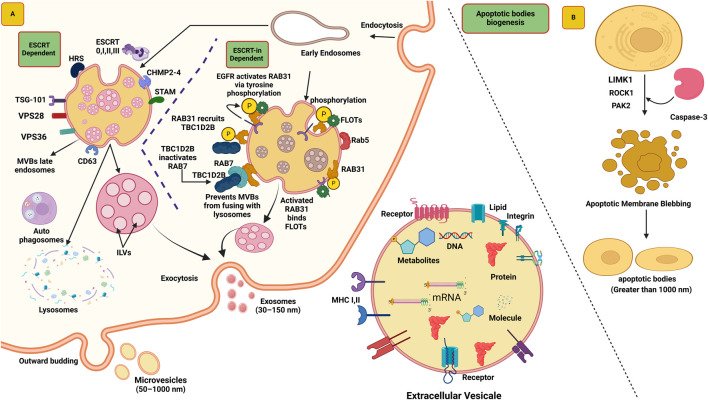
The biogenesis of exosomes depends on two pathways ESCRT dependent and ESCRT-independent. **(A)** The formation of exosomes begins with the inward budding of the plasma membrane, leading to the formation of early endosomes. Subsequently, the ESCRT complex (ESCRT 0, I, II, III) facilitates the formation of multivesicular bodies (MVBs). These MVBs either fuse with lysosomes for degradation (by autophagosomes) or translocate to the plasma membrane, where the SNARE complex aids their fusion, releasing the exosomes into the extracellular space. In ESCRT-independent mechanism RAB31 present on late endosomes that interact to EGFR, EGFR phosphorylate RAB31 viva tyrosine phosphorylation. Active RAB31 bind with Flotillin (FLOTs), now these complexes allow to formation of ILVs. Additionally, RAB31 recruit TBC1D2B that binds with protein RAB7 and prevents MVEs from fusing with lysosomes. **(B)** Biogenesis of apoptotic bodies begins with cytoskeletal reorganization and plasma membrane blebbing that regulated by ROCK1 and MLCK proteins. Caspase-3 triggers ROCK1, which then modifies myosin II, leading strong contractions of the cytoskeleton, and formation of membrane blebs. Further aspase-3 kinase cleaves PAK2 and add fatty acid to it, thereby increasing its activity, that help in cell membrane movement and controls cell shape. Moreover, activates JNK pathways, for the proper release of apoptotic EVs.

The ESCRT machinery is a multi-protein complex comprised of approximately 30 distinct proteins, which can be classified into four different complexes: ESCRT-0, ESCRT-I, ESCRT-II, and ESCRT-III. These protein complexes play crucial roles in the biogenesis of exosomes (Camacho et al., 2023). The investigation of ESCRT complexes has yielded significant insights into the intricate cellular mechanisms underlying the formation and release of exosomes, enhancing our understanding of intercellular communication and cellular trafficking processes (Ho et al., 2022). The biogenesis of ILVs begins by placing cargo on the outer surface of MVBs, and this action is facilitated by the ESCRT-0 complex. ESCRT-0 functions like a team management, capable of capturing the biomolecule utilized two crucial components: hepatocyte growth factor-regulated tyrosine kinase substrate (HRS) and signal transducing adaptor molecule (STAM). Both help the team to identify and attach to the cargo, preparing it for the subsequent stages in forming ILVs (Jin et al., 2022). The ESCRT-0 complex have ten binding sites that facilitate the capture of polyubiquitylated cargo (Dixson et al., 2023). Upon attachment of marked polyubiquitylated cargo to ESCRT-0, the HRS-STAM complex presents an opportunity for the ESCRT-I complex to participate in the process (Mirzaei et al., 2022). ESCRT-I, aided by the molecule TSG-101 (Tumor susceptibility gene 101), which binds to ubiquitin, facilitates the transport of the cargo (Mishra et al., 2023). Subsequently, ESCRT-I recruits the subsequent complex, ESCRT-II, through its interaction with VPS28 and VPS36 subunits (Hudait et al., 2023). ESCRT-II, in turn, recruits ESCRT-III, accompanied by the involvement of a specialized protein known as CHMP2-4 (Azad et al., 2023). Collectively, they form novel structures known as ILVs, thereby accomplishing the budding and cleavage of these diminutive vesicles from the membrane. Additionally, the accessory protein AAA-ATPase VPS4 performs a vital role in the disassembly and recycling the ESCRT-III complex (Tseng et al., 2022) (Figure 1A).

### 2.2 Biogenesis of exosomes through ESCRT-independent pathway

In mammalian cells, MVBs can still be synthesized even without ESCRT complexes, indicating that ILV biogenesis can happen independently of ESCRT. However, it has been noted that MVBs formed without ESCRT are larger and contain fewer ILVs with irregular shapes and sizes. RAB31 is a relatively small GTPase protein primarily responsible for intracellular trafficking and vesicle transport (Wei et al., 2021). The activation of RAB31 is crucial for exosome biogenesis, mediated through phosphorylation catalyzed by the EGFR (Horbay et al., 2022). After the activation of RAB31, it interacts with flotillin proteins of SPFH (Stomatin, Prohibitin, Flotillin, HflK/C) domain present in lipid raft microdomains (Wei et al., 2021). Flotillins are membrane-associated proteins that localize to specific cholesterol-rich microdomains in the plasma membrane known as lipid rafts (Lu and Fairn, 2018). The interaction between RAB31 and flotillin proteins facilitates the entry of EGFR into EVs, leading to the formation of ILVs (Lizarraga-Valderrama and Sheridan, 2021). Another role has been observed for this protein in EVs, similar to RAB31, which recruits TBC1D2B, deactivates RAB7, and prevents the fusion of EVs with lysosomes. This process creates a favorable environment for the formation of ILVs. Consequently, functional exosomes are released, playing a crucial role in intercellular communication and various cellular processes (Gao et al., 2022) (Figure 1A).

### 2.3 Biogenesis of microvesicles (MV) and apoptotic body

The MVs form when direct outward budding and subsequent shedding of the plasma membrane occurs (Clancy et al., 2021). In the formation of MVs, various factors work together, such as the redistribution of phospholipids and the contraction of the actin-myosin machinery (Abels and Breakefield, 2016). When looking into the detailed mechanism, ADP-ribosylation factor 6 (ARF6), a small GTPase protein, plays a significant role in the activation of phospholipase D (PLD) enzyme during its active state and leads to the repositioning of phosphatidylserine to the outer leaflet and initiates the process of MV formation (Menck et al., 2020). Additionally, the activity of ARF6 recruits the Extracellular Signal-Regulated Kinase (ERK) (mitogen-activated protein kinase (MAPK) family) to the plasma membrane. ERK’s presence is pivotal for downstream signaling pathways. Upon phosphorylation of ERK, it further activates myosin light chain kinase (MLCK). This activation leads to the phosphorylation of the Myosin Light Chain (MLC). The phosphorylation of MLC enhances the contractility of the actin-myosin complex, resulting in induced membrane curvature and budding. This activity ultimately leads to the genesis of small MVs from the plasma membrane. These vesicles contain specific biomolecules, such as ARF6, MHC-I, b1-integrin, VAMP3, and MT1MMP (Abels and Breakefield, 2016).

Apoptotic bodies also a membrane-bound structure that also contain variety of cargos such as proteins, lipids, RNA, miRNAs and DNA, that involve in intercellular communications. The feature of apoptotic bodies can vary depending on the cell type (Santavanond et al., 2021). There is not much information available about the role of apoptotic bodies in cancer biology, mostly scientific work is being done utilizing exosomes and microvesicles. The biogenesis of apoptotic bodies begins with cytoskeletal reorganization and plasma membrane blebbing. Previously, it was believed that this process occurred randomly, but recently it was recognized that the biogenesis of apoptotic bodies occurs through well-ordered morphological steps. Rho-associated coiled-coil-containing protein kinase 1 (ROCK1) and myosin light chain kinase (MLCK) regulates biogenesis of apoptotic bodies (Phan et al., 2020). In this mechanism, caspase-3 activation triggers ROCK1, which then modifies myosin II, resulting in strong contractions of the cytoskeleton, leading to the formation of membrane blebs. It is not yet completely clear which MLCK is active, but inhibition of MLCK has been shown to prevent membrane blebbing. Additionally, another protein, LIMK1, helps in this process by activating cofilin that also regulates the actin skeleton and supports apoptotic membrane blebbing (Sebbagh et al., 2001). Caspase-3 kinase cleaves p21-activated kinase 2 (PAK2) adds a fatty acid to it, thereby increasing its activity. Active PAK2 helps in cell membrane movement and controls cell shape. It also activates signaling pathways such as JNK, which play a role in proper release of apoptotic EVs (Han et al., 2024) (Figure 1B).

## 3 Role of EVs in TNBC

TNBC is a type of aggressive and refractory BC mainly occurs in young patients and has a poor clinical prognosis. So far, no specific target has been identified for its management on which intervention can be done. To achieve better treatment outcomes, a promising drug is needed, and in recent years EVs have shown significant promise in the management of TNBC (St-Denis-Bissonnette et al., 2022). In a study conducted by Ozawa et al., it was revealed that EVs originating from the HCC1806 malignant cell line possess remarkable capabilities. These EVs not only promote the spread of TNBC tumors in the non- malignant MCF10A cell line but also play a significant role in inducing drug resistance, thereby enhancing the survival of the recipient cells. Additionally, these EVs display an exceptional ability to modulate specific miRNAs intricately linked to tumor-related processes (Ozawa et al., 2018). EVs originating from TNBC tumor cells have been observed to substantially impact tumor development and metastasis. Notably EVs release from TNBC cells line (HCC1806) can enhance the growth of normal mammary epithelial cells (MCF10A), Additionally induce drug resistance by activating PI3K/AKT, MAPK and HIF1α signaling pathways (Das K. et al., 2023). EVs have multifaced role including the facilitation of tumor proliferation, angiogenesis, and immune system evasion. These effects are achieved through the targeted delivery of specific biomolecules to adjacent cells, thereby modulating their behavior and providing crucial support for neoplasm progression (Tai et al., 2018). EVs derived from TNBC cells transfer oncogenic proteins such as EGFR and MMPs (Matrix metalloproteinases) to recipient cells, that enhancing their invasiveness and migratory traits (Mashouri et al., 2019).

EVs can modulate the TME in a manner that facilitates the development of pre-metastatic sites. Specifically, these EVs can instruct stromal cells, such as fibroblasts and immune cells, to create a supportive and conducive environment for the proliferation and dissemination of the tumor cells (Liu et al., 2020). Additionally, EVs have the remarkable capacity to induce modifications in gene expression and several signaling pathways within recipient cells, subsequently resulting in profound alterations in their phenotypic characteristics and functional role (Mashouri et al., 2019).

### 3.1 EVs in TNBC prognosis

Comparative analysis of plasma samples between healthy and patients suffering from TNBC, a conspicuous presence of small extracellular vesicle (sEVs) has been discerned. Specially, these sEVs are substantially enriched in TNBC patients (Stevic et al., 2018). Additionally, a distinct dissimilarity has been noticed in the expression patterns of sEV-miRNA between TNBC and HER2-positive patients. Notably, miR-335, miR-422a, and miR-62 have emerged as specific examples of such differential expression. In the context of TNBC, miR-374, which is linked to sEVs, plays a crucial role in promoting increased tumor size (Zhou et al., 2022). In contrast, several other miRNAs, namely, miR-185, miR-376a, miR-382, miR-410, miR-433, and miR-628, displayed an association with HER2-positive patients (Liu T. et al., 2021). The substantial secretion of sEVs depends upon the upregulation of the TSAP6 protein (Negahdaripour et al., 2021). Functionally, TSAP6 maintains cellular homeostasis and impedes carcinogenesis (Pavlakis et al., 2020). In the context of DNA damage, the activation of the p53 protein subsequently induces the transcription of TSAP6 (Nagao et al., 2022).

The SKBR3 cell line exhibits a high proliferative rate and demonstrates elevated expression levels of the Her2 (Neu/ErbB-2) gene product (Coppola et al., 2022). In contrast, the MDA-MB-231 cell line represents a poorly differentiated TNBC cell line characterized by the absence of ER and PR expression. Similarly, the HCC1954 cell line also overexpresses Her2/neu (Shiravi et al., 2021). Upon the reception of sEVs derived from TNBC, a significant enhancement is observed in the proliferation, migration, and invasion capacities of these cell lines (Zhou et al., 2022). Wills et al. conducted a study on tumor metastasis mechanisms facilitated by EVs post-chemotherapy. For this they used xenograft mouse models of TNBC, and notice that Doxorubicin increased the release of sEVs from malignant cells, thereby promoting pulmonary metastasis. Utilizing proteomic analysis and CRISPR/Cas9 gene editing, they identified glycoprotein Pentraxin 3 (PTX3) as abundant in Doxorubicin-induced sEVs (Wills et al., 2021). PTX3 can trigger the NF-κB pathway, which is a key regulator of tumor cell proliferation and survival (Rathore et al., 2019). Consequently, PTX3 plays a pivotal role in regulating chemotherapy-induced metastasis and chemoresistance, thereby suggesting it as a potential therapeutic target against the adverse effects of chemotherapy on metastatic progression and chemoresistance (Wills et al., 2021).

### 3.2 EVs in diagnosis of TNBC

In diagnosing TNBC, imaging and immunohistochemistry (IHC) are the two primary tools currently being used (Roostee et al., 2023). Imaging tools identify TNBC by detecting BC masses or other irregularities. Frequently utilizing imaging tool for TNBC include mammography, ultrasound, and magnetic resonance imaging (MRI) (Sha and Chen, 2022). Mammograms can detection oncological diseases, but less effective in the case of TNBC when compared to other types of malignancy. This limitation is because of TNBC lacking distinctive features such as speculated margins or microcalcifications commonly found in different pathology (Chen and Lee-Felker, 2023). Due to this reason, it becomes quite challenging to identify it through mammograms. Firstly, it may yield false-negative results, as it cannot provide 100% accurate information about TNBC. It may miss one in eight TNBC cases, particularly in women with dense breast tissue, leading to a false sense of reassurance (Gegios et al., 2023). Secondly, false-positive results may occur, where in a positive result is shown even in the absence of disease. This is more prevalent in younger women, those with dense breasts, those who have previously undergone breast biopsies, have a family history of BC, or women taking estrogen, and so on (Wong et al., 2023).

Ultrasound is a beneficial tool for the detection of TNBC (Wang and Wang, 2023), playing an important role in determining the patient’s condition. Ultrasound is significantly superior to mammography as it can easily identify small, non-calcified lesions, accurately differentiate between solid and cystic lesions, and reduces false positive results (Emory et al., 2023). In a study, it was found that the sensitivity of ultrasound for detecting TNBC ranges from 60%–80%, and the specificity ranges from 70%–90%. In spite of these findings, some limitations are associated with it (Ahmed, 2018). Ultrasound has a lower susceptibility to detect TNBC compared to other types of BC. In addition, it is not as effective as other imaging modalities in the accurate staging of TNBC (Dogan and Turnbull, 2012). These features contribute to the challenges in identifying TNBC and determining its extent using ultrasound imaging.

MRI a highly sophisticated tool for detecting TNBC, and it is remarkably better than mammography or ultrasound techniques (Kong et al., 2022). MRI is proficient in precisely identifying tiny tumors. MRI can also determine the location, size of TNBC, and it can also detect the spread of the tumor (Taourel et al., 2018). MRI also serves as critical role in monitoring the progress of TNBC management. Additionally utilized in planning the surgery for tumor removal (Ross and Chenevert, 2021). Despite its advantages, MRI has some limitations compared to mammography and ultrasound (Ma et al., 2022). First of all, it is significantly more expensive than imaging tools. Secondly, it is not as widely available as other imaging options. Additionally, it requires more time to perform and can sometimes be uncomfortable for patients (Ormond Filho et al. 2019).

Biopsy another technique which are done for TNBC. In this procedure, a small piece of breast tissue is extracted and examined under the microscope, and provides significant information about TNBC and its grade (Beňačka et al., 2022). One of the major diagnostic techniques performed on biopsy specimens is immunohistochemistry (IHC). This is a diagnostic test that relies on specific antibodies that specifically detect specific types of proteins or molecules on the surface of tumor cells. For instance, in the case of TNBC, it is performed based on the expression of ER, PR, and HER2 (Wang et al., 2022). IHC also identifies specific proteins or biomolecules that are expressed in TNBC, such as EGFR (epidermal growth factor receptor), Ki-67 (a proliferation marker), and p53 (a tumor suppressor gene) (Lu et al., 2023). This test is extremely beneficial because of its quick results and its heightened sensitivity in detecting TNBC. It can also identify molecules that significantly contribute to the aggressiveness and metastasis potential of TNBC (Ribeiro et al., 2022), and also provide significant assistance in intervention. The result this test may vary depending on the laboratory that performs the test (Cardos et al., 2022). Additionally, IHC cannot diagnose all cases of TNBC because not all cases show the presence of ER, PR, and HER2. These are some limitations that are specific to the IHC test.

Mutations in genes identify by genetic testing that provide crucial information regarding malignancy for instance BRCA1 and BRCA2 in BC. Mutations in these genes are strongly associated with the development of BC and help confirm risk as well as guide treatment (Ponti et al., 2023). Molecular profiling techniques can provide insights into the biological characteristics of TNBC and significantly aid in its therapy (Das D. et al., 2023). The majority of patients with TNBC often receive their diagnosis at an advanced stage because biomarkers that can effectively detect the tumor are absent during primary stage of TNBC (Das D. et al., 2023; Ponti et al., 2023). In order to streamline and enhance TNBC treatment, it becomes important to discover biomarkers as early as possible to detect TNBC in its early stages (Agostinetto et al., 2022). By identifying such biomarkers, the diagnosis and subsequent strategy of TNBC can be simplified and made more accessible, potentially improving patient outcomes and overall prognosis. Researchers anticipate that EVs will play a crucial role in the diagnosis and treatment of a variety of diseases in the future. EVs can carry oncogenic proteins, which may provide important insights into cancer initiation, progression, risk assessment, and treatment strategies (Wang J. et al., 2019).

#### 3.2.1 EVs-associated proteins in TNBC diagnosis

The markers present on the surface of EVs derived from TNBC, along with the proteins packaged within them can greatly help in the early estimation of an aggressive neoplasm diagnosis (Dong et al., 2022). Various methods are available for EVs isolation from different sources such as urine, plasma, serum, MSCs and other body fluids, but most widely accepted are ultracentrifugation (Giovanazzi et al., 2023). These EVs are further confirmed by various techniques such as DLS (dynamic light scattering), nanoparticle tracking analysis (NTA) and Electron microscopy for their size, and Western blotting using specific EVs markers such as tetraspapnin, CD63, and TSG and annexin IV. Furthermore, the flow cytometry is the best methods to quantify the specific proteins associated with EVs utilizing specific antibodies against them (Zhang et al., 2021; Tiwari et al., 2024).

##### 3.2.1.1 EVs associated EGFR (epidermal growth factor receptor) in TNBC diagnosis

**FIGURE 2:**
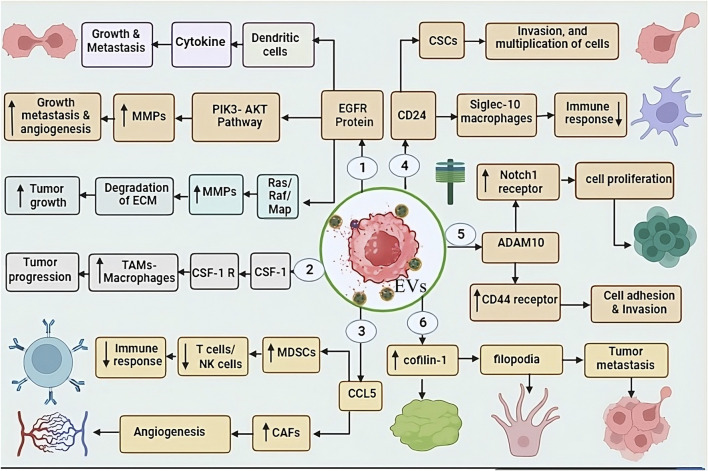
This figure shows the role of EV-associated proteins in TNBC progression through various molecular interactions: 1. EGFR-EVs: promoting tumour growth and metastasis through dendritic cells and cytokines. EGFR activates the PI3K-AKT pathway, upregulating matrix metalloproteinases (MMPs), leading to tumour invasion, metastasis, and angiogenesis. Additionally, EGFR also activates the Ras/MAPK pathway, increasing MMP activity, resulting in ECM degradation and tumour progression. 2. CSF1-EVs: target tumour-associated macrophages (TAMs), stimulating their activity and enhancing tumour progression. 3. CCL5-EVs enhance the function of MDSCs and CAFs. MDSCs suppress T cell/NK cell activity, leading to immune evasion, while CAFs promote angiogenesis. 4. CD24-EVs: Suppress immune response by interacting with Siglec-10 on macrophages, thereby helping TNBC cells escape immune surveillance. 5. ADAM10-EVs: Regulate cell proliferation and adhesion by targeting Notch-1 and CD44 receptors, which contribute to tumour progression. 6. Cofilin-1-EV: Enhances tumour metastasis, further increasing the aggressiveness of TNBC.

EGFR is a trans-membrane glycoprotein present on the cell surface, after binding of epidermal growth factor molecule, it initiates intracellular signaling cascades. Resulting regulates various cellular processes including proliferation and differentiation, thereby controlling cell growth, division and cell division (Sabbah et al., 2020). The overexpression of EGFR has been identified as a contributing factor in several cancers, including TNBC (Hsu and Hung, 2016). Previous studies have revealed that EGFR protein is found on the surface of EVs secreted by TNBC cells, plays a crucial role in its propagation, dissemination, growth and metastasis (St-Denis-Bissonnette et al., 2022), and it utilizes various methods to carry out these functions (Zakaria et al., 2019). For instance, EVs displaying EGFR transfer EGFR to immune cells, such as dendritic cells. As a result, dendritic cells become activated and start producing pro-inflammatory cytokines. Consequently, this leads to the growth and metastasis of tumor cells (Frawley and Piskareva, 2020). It also participates actively in cell proliferation by triggering crucial pathways such as EGFR, Ras-Raf-MAPK, and PI3K-Akt pathways. It can facilitate the degradation of the extracellular matrix by promoting matrix metalloproteinases (MMPs), resulting metastasis. Additionally, involved in angiogenesis and resistance to therapy (Dey et al., 2022). The varied functions of EGFR confer its strong potential for clinical translation in both discernment and therapeutic interventions for TNBC (Figure 2).

##### 3.2.1.2 EVs associated CSF 1 (colony-stimulating factor 1) in TNBC diagnosis

The EVs associated protein CSF-1 have pivotal role in recruiting and polarizing tumor-associated macrophages (TAMs). CSF-1 can significantly enhance the tumor growth, invasion, and metastasis, that is key factor for TNBC development (Cannarile et al., 2017). For this, CSF-1 behaves like a cytokine. It binds to its receptor, colony-stimulating factor 1 receptor (CSF1R), which is present on the surface of TAMs. This interaction triggers specific signaling transduction, leading to the recruitment and differentiation of TAMs (Muñoz-Garcia et al., 2021). CSF-1 and TAMs are prominently present in the case of TNBC, contributing to tumor progression and creating an environment characterized by immunosuppression and pro-tumorigenic factors (Baig et al., 2020). consequently, CSF-1 can play a importent role as a crucial biomarker in the diagnosis of TNBC, which will be highly valuable in its therapeutic approach (Kuemmel et al., 2022) (Figure 2).

##### 3.2.1.3 EVs associated CCL5 (chemokine ligand 5) in TNBC diagnosis

CCL5 is also identify as RANTES, and its key function is to attract immune cells such as T cells, B cells, and natural killer (NK) cells (Mgrditchian et al., 2017). It interacts with TME-derived EVs, and has been associated with poor prognosis in numerous types of neoplasm, including TNBC (St-Denis-Bissonnette et al., 2022). In TNBC, CCL5 attracts immune cells that participate in suppressing the immune response, such as myeloid-derived suppressor cells (MDSCs), which inhibit the activity of T cells and NK cells (Weber et al., 2021). Additionally CCL5 promote the growth of CAFs, that is responsible for tumor growth and metastasis (Mao et al., 2021). CCL5 has the ability to induce the formation of angiogenesis cells, which are necessary for tumor growth, nourishment, and dissemination, and it attracts endothelial cells, the lining cells of blood vessels, to carry out this function (Do et al., 2020). In a study conducted on rats with TNBC, it was observed that if CCL5 is blocked using antibodies can significantly reduce tumor metastasis, and it was also observed that the number of T cells and NK cells increased significantly. CCL5 emerges as a potential predictive biomarker for assessing the risk of pathology recurrence in TNBC patients. Empirical investigation has demonstrated a significant association between elevated CCL5 levels in tumor specimens and an augmented susceptibility to neoplasm relapse within a 5-year period following therapeutic intervention (Figure 2).

##### 3.2.1.4 EVs associated cluster of differentiation 24 (CD24) in TNBC diagnosis

The GPI-anchored protein CD24-EVs are identified in various biological fluids of cancer patients and serve as a marker of EVs. IHC showed that CD24^+^ EVs were detected in the serum of melanoma patients and BC, and it is also known as the heat-stable antigen CD24 (Gilliam et al., 2017). CD24 is an extremely small cell surface protein characterized by extensive glycosylation and its linkage to the glycosylphosphatidylinositol on the cell surface (Altevogt et al., 2021). It finds expression in various cells, including B cells, T cells, neutrophils, and epithelial cells, among others (Altevogt et al., 2021). Its primary function is to take responsibility for various cellular processes such as cell adhesion, migration, differentiation, and apoptosis (Shirmohamadi et al., 2020). CD24 also plays a crucial role in the tumor growth, cellular proliferation, epithelial-mesenchymal transition, angiogenesis, invasion, metastasis, promoting Immune invasion and acquisition of drug resistance in TNBC (Altevogt et al., 2021). It behaves like an anti-phagocytic surface protein and sends a “do not eat me” signal to immune cells like macrophages, discouraging them from attacking or engulfing the tumor cells (Barkal et al., 2019). Along with that, it interacts with Siglec-10 protein present on the surface of macrophages that exhibits resistant to tumor cells. This interaction significantly reduces CD24’s inhibitory capacity against tumors, Subsequently impairing the ability of immune system to combat tumors (Zhao et al., 2023). Moreover, CD24 is found to be overproduced in pathologically stem cells (CSCs), highly specific cells within tumors with the ability to self-renew and initiate tumor growth (Liu et al., 2014). As per findings from investigations in TNBC, CD24 emerges as a highly potential promising biomarker, given its crucial role in the disease (Chan et al., 2019) (Figure 2).

##### 3.2.1.5 EVs associated ADAM10 in TNBC diagnosis

ADAM10 that is member of Metalloproteinase (ADAM) and disintegrin family (Matthews et al., 2017), exhibits expression in various tissues. It is a multifaceted enzyme that can cleave verity of proteins, including cell surface receptors, extracellular matrix proteins, and other ADAMs (Shimoda et al., 2021). ADAM10 have pivotal role in the etiology of diverse disease, including TNBC (Cheng et al., 2021). EVs associated ADAM10 can initiate the activation of Notch signaling transduction by inducing the expression of the Notch1 receptor (Alabi et al., 2021), a process associated with cellular proliferation, enhancing migratory potential. In addition, it also increases the expression of CD44 receptor, which significantly contributes to cell adhesion and invasion. Due to the significant increase in CD44 expression in TNBC, the disease spreads to different body parts (Alabi et al., 2021; Cheng et al., 2021). ADAM10-EVs confer resistance to therapeutic interventions, such as chemotherapy in tumors cells (Tan et al., 2018) (Figure 2).

##### 3.2.1.6 EVs associated Cofilin-1 in TNBC diagnosis

Cofilin family protein like Cofilin-1, is responsible for cell motility and actin depolymerization (Tahtamouni et al., 2022). Its crucial role in various types of disease has been well-documented, with particularly elevated expression levels observed in EVs released from TNBC (Howard et al., 2022). The actin protein is fundamentally essential for cellular motility and cytoplasmic spreading, with its regulation primarily under the control of Cofilin-1 (Sousa-Squiavinato et al., 2019). In the context of TNBC, the overexpression of Cofilin-1 assumes a critical function in facilitating tumor infiltration and metastatic dissemination (Howard et al., 2022). Cofilin-1 promotes metastasis in TNBC by aiding actin-rich filopodia (Aseervatham, 2020). Filopodia a finger-like projections that extend from the surface of cells and have the ability to adhere to their surrounding environment (Jarsch et al., 2020). Cofilin-1 promotes tumor cell spread by weakening cell connections, enabling them to break away from the primary tumor (Zhang et al., 2018). Additionally, it also helps cancer cells survival by preventing apoptosis, as higher level of cofilin-1 have been linked to resistance against chemotherapy and radiation therapy, both of which rely on apoptosis to kill cancer cells (Chen et al., 2020; Howard et al., 2022) (Figure 2).

##### 3.2.1.7 EVs associated non-coding RNAs in TNBC diagnosis

Non-Coding RNAs (ncRNAs) derived through EVs, such as miRNA, lncRNA, and circRNA, have been extensively studied in the context of various tumors. miRNA, renowned for its versatility and multifaceted regulatory roles in various cellular processes, including cell signaling, homeostasis, and cell fate. It can also function as tumor suppressors or oncogenes for this purpose (Rupaimoole and Slack, 2017). The search for reliable tools for diagnosing different subtypes of BC at the molecular level has always been the focus of research efforts. miRNA has emerged as highly capable of achieving accurate diagnosis (Yang et al., 2019). Several studies have indicated that by analyzing the expression patterns of different miRNAs, can distinguish between BC samples and normal tissues, as well as differentiate TNBC from other types of clinical BC (Sabit et al., 2021).

The researchers examined plasma EVs of TNBC patients and healthy individuals, identifying 20 upregulated and 34 downregulated miRNAs in these EVs compared to healthy controls. Among the upregulated miRNAs, miR-150-5p and miR-4665-5p demonstrated the ability to differentiate TNBC patients who respond positively to therapy and those who do not. This discovery has led scientists to believe that this could be an unprecedentedly valuable tool for the diagnosis and potential management of TNBC (Ozawa et al., 2020). In a study, the researchers identified four EV-associated miRNAs: miR-142-5p, miR-150-5p, miR-320a, and miR-4433b-5p. After analyzing these miRNAs, they created a miRNA profile consisting of miR-142-5p, miR-320a, and miR-4433b-5p, which could differentiate between TNBC patients and healthy individuals. The sensitivity of this profile was 93.33%, and the specificity was 68.75%. moreover, the reduced expression of miR-142-5p and miR-150-5p in patients indicated a high advanced stage of tumor classification (Carvalho et al., 2022). In clinical practice, multiple studies have unveiled insights into the utility of serum EV-miRNA as a targeted indicator to predict the efficacy of diverse prevention strategies in TNBC. Such studies shed light on the pivotal clinical application of EV-miRNAs as specific biomarkers. One such investigation was conducted during a randomized phase II neoadjuvant trial known as Geparsixto (Stevic et al., 2018).

Long non-coding RNAs (lncRNAs) are important and specific components of the genetic program that modulate tumor cells and can influence their characteristics, exhibiting role in mediating tumor initiation and progression (Hu Q. et al., 2020). The lncRNA transcript panel exhibits an association with normal breast tissue, TNBC, and its subtypes. Through comprehensive analysis of transcriptome, molecular classification of BC becomes possible. Thereby, facilitating the identification and diagnosis of specific molecular signatures exclusively related to TNBC (Rodríguez Bautista et al., 2018). SUMO1P3 is a type of lncRNA that exhibits peculiar behavior in various cancer types, especially when found in high amounts in blood-derived EVs. It is strongly associated with negative prognosis and ineffective treatment outcomes in TNBC patients compared to individuals without disease or those in good health (Hu et al., 2021). In a recent study, it has been elucidated that the expression level of exosome lncRNA XIST decreases significantly after surgical resection of the primary breast tumor. However, upon the recurrence of BC, a notable and statistically significant elevation in the expression level of exosomal lncRNA XIST is observed. Consequently, EVs lncRNA XIST holds promise as a robust biomarker for patients with recurrent TNBC. Especially, this predictive ability remains independent of confounding variables associated with the patient’s clinical and pathological condition (Lan et al., 2021).

### 3.3 Role of artificial intelligence (AI) in TNBC diagnoses

AI, especially with advances in deep learning (DL), a subset of machine learning (ML), has made significant contributions to addressing various clinical challenges in oncology, including tumor diagnosis, intervention, and prognosis. DL has the ability to automatically extract big data and process it, which has revolutionized areas such as image classification, neural language processing, and audio/video analysis. AI application in medical imaging has made diagnosis more accurate while reducing false positives, demonstrating its transformative potential in improving healthcare outcomes (Liao et al., 2023). DL exhibited significant success in the diagnosis of various types of pathology, like liver, colorectal, prostate, and BC, by using latest imaging modalities such as MRI, mammography, ultrasound (US), computed tomography (CT), and positron emission tomography-CT (PET-CT), DL plays an important role in increasing diagnostic accuracy and reliability, thereby improving oncology care and improving patient outcomes.

Firstly, any effective therapeutic approach for TNBC pathology, early screening and diagnosis is essential. While MRI is potential to effectively differentiate between TNBC subtypes, but there is a need to determine its various stages. Prognostic challenges arise due to heterogeneous predictive biomarkers, making predictions more complex. AI has significantly improved TNBC diagnosis at all stages through advanced algorithms, increasing both accuracy and efficiency. Integrating AI into screening programs has led to substantial improvements in clinical outcomes (Batool et al., 2024). AI integration with spectroscopic techniques such as Raman spectroscopy has significantly increased TNBC prediction accuracy, achieving rates up to 96.7% (Leithner et al., 2020). In TNBC pathology, need to critical diagnostic biomarkers to guide immunotherapy and prognosis. AI has proven to be a valuable tool in this domain. In a recent study by Li et al. developed an immune infiltration cell (IIC)-associated signature (MLIIC) for TNBC using transcriptomic data from purified immune cells, TNBC cell lines, and patient samples, as well as 25 machine learning algorithms including Boruta, LaSolR, SVM, and XGBoost. The results identified IIC-related RNAs (IIC-RNAs) using the tumor-stroma index (TSI), which displayed different expression patterns—upregulation in immune cells and downregulation in TNBC cells. The MLIIC signature demonstrated strong predictive value, correlating with survival outcomes. Its significance was further validated through immunofluorescent staining, confirming its potential as a reliable biomarker for TNBC prognosis (Li et al., 2023). As mentioned earlier, lack of these receptors (ER, PR, and HER2) renders many standard treatments ineffective. AI has the potential to use molecular and genetic data to identify therapeutic targets and predict strategy responses. Its integration could significantly enhance precision medicine approaches and improve treatment outcomes in all TNBC stages (Tsou et al., 2020). Similarly, the ML algorithm IDtrax holds promising potential in identifying specific therapeutic targets for TNBC, enabling targeted drug development and personalized therapy. Additionally, it is able to predict effective inhibitors (Gautam et al., 2019).

### 3.4 EVs in developing drug resistance in TNBC

#### 3.4.1 EVs role in resistance to EGFR targeted therapy for TNBC

TNBC cells secrete a multitude of cargo components within EVs, which play a significant role in conferring drug resistance in TNBC. These cargo components encompass drug efflux-promoting proteins, oncogenic molecule, and biomolecule capable of modulating signaling pathways (Maleki et al., 2021). Notably, TNBC exhibits significantly high expression of EGFR (Zoeller et al., 2020), and these tumors inherently possess resistance to EGFR inhibitors (EGFRi), EVs also play crucial role for this like EVs encapsulated EGFR are protected from the inhibitory effect of EGFRI, such as Erlotinib, Gefitinib (reversible inhibitors), Afatinib (irreversible inhibitor), Bendamustine and osimertinib. Additionally, the transfer of EGFR through TNBC-EVs can activate signaling pathways in recipient TNBC cells, leading to resistance against EGFRi. Overcoming EGFRi resistance may be an alternative option for the management of TNBC (Costa et al., 2017; Hung et al., 2019).

**FIGURE 3:**
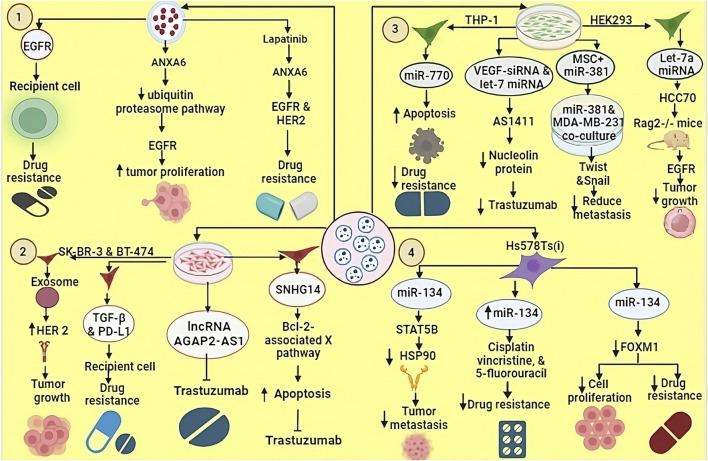
EVs derived from TNBC cells line can transfer various biomolecules to recipient cells, thereby modulated their biological functions. 1. EVs carrying EGFR contribute to drug resistance in recipient cells. ANXA6-containing EVs inhibit the ubiquitin-proteasome pathway, leading to EGFR-mediated tumour growth and proliferation. 2. EVs secreted by SK-BR-3 and BT-474 cell lines transport bioactive molecules such as TGF-beta, PD-L1, lncRNA AGAP2 and SNHG14, which promote tumour growth and resistance to trastuzumab. 3. miR-770 derived from THP1 cells enhances apoptosis and counteracts drug resistance. VEGF-derived siRNA and let-7 miRNA target AS1411, which inhibits nucleolin proteins, thereby increasing drug sensitivity. Let-7a miRNA, when loaded into EVs and introduced into TNBC HCC70 cells, was tested in Rag2^−/−^ mice resulting Let-7a miRNA suppresses tumor growth by blocking the EGFR signaling pathway. 4. EVs from Hs578Ts cells carrying miR-134 reduce metastasis by inhibiting HSP90 activity, increase sensitivity to cisplatin, vincristine, and 5-fluorouracil, and inhibit tumor cell proliferation.

TNBC is primarily managed through systemic chemotherapy, which remains the mainstay of treatment (Luo et al., 2022). One of the highly efficacious drugs used in TNBC is Gemcitabine, also referred to as dFdC (2′,2′-difluorodeoxycytidine) (Zhao et al., 2020). Gemcitabine demonstrates notable effectiveness specifically in TNBC patients who have previously undergone regimen with anthracyclines and taxanes (Li et al., 2021). In cases of metastatic TNBC, the combined administration of platinum-based agents and gemcitabine provides substantial benefits (Pandy et al., 2019). The utilization of this combination therapy demonstrates significant efficacy in managing TNBC metastasis. However, TNBC cells exhibit remarkable capacity to develop rapid and efficient drug resistance (Zhang et al., 2022). Extensive research has revealed that these drug-resistant TNBC cells possess the capability to transfer their resistance to sensitive cells via EVs. This phenomenon allows the transmission of acquired drug resistance within the TNBC cellular population (Xavier et al., 2022). TNBC cells that have developed resistance to gemcitabine exhibit heightened expression levels of Annexin A6 (ANXA6) within both the cellular and EV compartments (Yi, 2023). ANXA6, functioning as a calcium-dependent membrane-binding protein, imparts resistance to multiple drug classes used in TNBC therapy (Noreen et al., 2020). Mechanistically, ANXA6 engages in interactions with EGFR and impedes the ubiquitin-proteasome pathway, thereby facilitating the accumulation of active EGFR and fostering tumor proliferation and dissemination (Li et al., 2021). Lapatinib, a bivalent inhibitor targeting both **Vesicle-associated Proteins in TNBC** and HER2, exhibits the capacity to counteract the resistance mediated by EVs ANXA6 (EV-ANXA6). Moreover, the circulating ANXA6 levels in the serum of TNBC patients serve as prognostic indicators for the responsiveness to gemcitabine-based chemotherapy. As previously stated, monotherapy with EGFR inhibitors proves inadequate for TNBC treatment due to the inherent resistance to EGFRi (Yi, 2023) (Figure 3).

#### 3.4.2 EVs role in resistance to human epidermal growth factor receptor 2 (HER2)-targeted therapy in TNBC

The persistent over-expression of HER2 in BC instances has posed an enduring and significant prognostic dilemma (Lee et al., 2015). However, the advent of targeted therapies aimed at HER2 has led to remarkable achievements in the effective management of BC (Simmons et al., 2022). Trastuzumab, as the initial monoclonal antibody targeting HER-2, has gained approval for the remedy of HER2-positive BC, imparting significant enhancements to long-term survival and disease-free maintenance (Jagosky and Tan, 2021). Nevertheless, despite the promising initial outcomes, studies have shown that resistance to HER2-targeted drugs develops in the majority of patients after approximately 1 year of therapeutic approach (Derakhshani et al., 2020). Several studies have demonstrated that EVs exhibit interference with the efficacious functioning of trastuzumab, resulting in neutralization of its effects. EVs were obtained from SK-BR-3 and BT-474 cell lines, which revealed substantial upregulation of HER2 expression within these EVs. Similar findings were observed in experimental analyses involving samples from BC patients, which also revealed a significant abundance of HER2 (Dong et al., 2020).

In a study conducted by Martinez et al., it was discovered that HER2-targeted drugs, which eventually develop resistance in HER2-negative cells are modulated by two pivotal biomolecules: transforming growth factor beta 1 (TGF-β1) and programmed death-ligand 1 (PD-L1). Notably, the researchers observed the transfer of TGF-β1 and PD-L1 from resistant cells to sensitive cells via EVs. This process plays a critical role, and these transferred molecules exert an inducible effect, causing the acquisition of characteristics exhibited by the source cells within the sensitive cells (Martinez et al., 2017). The levels of TGF-β1 associated with EVs are related to the response of patients with HER2+ BC to HER2- targeted strategy, suggesting TGF-β1 could potentially be used as a biomarker to assess the management effectiveness.

Dysregulation of non-coding RNAs also plays a crucial role in developing resistance to trastuzumab in BC (Singh et al., 2022). Trastuzumab-resistant cells exhibit the release of EVs containing a specific long non-coding RNA known as SNHG14. This SNHG14 RNA molecule, in turn, induces resistance to trastuzumab by impeding the process of cell apoptosis through the modulation of the B cell leukemia/lymphoma 2 (BCL-2)/Bcl-2-associated X pathway (Lampropoulou et al., 2022). Moreover, a notable observation reveals significantly elevated expression of SNHG14 in trastuzumab-resistant cells, while its expression remains minimal in cells sensitive to trastuzumab (Shen et al., 2021). Similarly, the lncRNA, i.e., AGAP2-AS1 has the potential to induce resistance to trastuzumab in BC cells (Zheng et al., 2019). The protein heterogeneous nuclear ribonucleoprotein (A2/B1) hnRNPA2B1 is implicated in the process of packaging lncRNA AGAP2-AS1 into exosomes (Zheng et al., 2019). However, as of now, comprehensive information about the precise molecular mechanisms by which this resistance is conferred remains elusive. Further investigations are required to elucidate the underlying mechanisms responsible for the development of trastuzumab resistance mediated by exosomal lncRNA AGAP2-AS1 in BC cells (Dong et al., 2020) (Figure 3).

#### 3.4.3 Role of EVs-associated miRNA in drug resistance

The miRNAs found in TNBC-EVs can cause drug resistance by influencing several processes (Li et al., 2020). A crucial protein called Protease-activated receptor 2 (PAR2) plays a role in producing and releasing EVs (Sung et al., 2019), and these EVs, through various signaling pathways like the AKT/NF-κB pathway, can transform normal cells into malignant cells. Moreover, these EVs carry specific miRNAs, such as miR-221, which regulate gene expression by reducing the mRNA expression of phosphatase and tensin homolog (PTEN) through targeting their 3′-untranslated region (3′-UTR) (Yi, 2023). The sEVs derived from MDA-MB-231 cells induce cellular resistance against Cisplatin, a chemotherapeutic agent used in the intervention of TNBC (Wang B. et al., 2019). Cisplatin exerts its cytotoxic effect by forming covalent cross-links between adjacent DNA strands, thereby inhibiting the process of DNA replication and transcription, ultimately triggers apoptosis (Tchounwou et al., 2021).

### 3.5 EVs in TNBC treatment

Recent advancement in targeted therapy and immunotherapy are playing an important role in the management of TNBC, such as inhibitors of key pathways (PI3K/AKT/mTOR and Notch), as well as immune checkpoint inhibitors like pembrolizumab. These approaches offer promising strategies for personalized therapeutic approach of TNBC patients. Such precise targeting is helpful in improving TNBC management and guiding future therapeutic approaches (Zhu et al., 2023). EVs play a key role in TNBC management (Brena et al., 2022). They contribute to drug resistance through paracrine signaling to nearby cells or by affecting the whole body, thereby hindering satisfactory treatment outcomes (Xavier et al., 2022). EVs also play a role in developing and strengthening new drug resistance (Grimaldi et al., 2023). However, they possess essential qualities such as specific targeting, immune compatibility, low toxicity, and a protective layer, making them an excellent option for delivering diverse drugs or biological molecules to treat tumor (Khosravi et al., 2022). In a research investigation on exosomes derived from THP-1 cells, the presence of the biomolecule miR-770 was identified (Liu J. et al., 2021). It was observed that miR-770 plays a role in promoting apoptosis and reducing chemotherapy resistance miR-770 targets STMN1 to enhance chemo-sensitivity and suppress metastasis. To explore the underlying molecular machinist Y. Li et al. using three bioinformatics tools—TargetScan, miRDB, and PICTAR5—identified predicted binding sites for miR-770 at positions 54–60, 267–273, and 409–415 in the 3′ UTR region of the STMN1 gene. Additionally, clinical evidence indicated a positive correlation between elevated miR-770 expression in cells or tissues and a notable enhancement in drug sensitivity. Consequently, these findings propose the potential utility of exosomal miR-770 as a promising therapeutic target or a prognostic indicator for TNBC (Li et al., 2018).

Experiments employing siRNA-loaded EVs have revealed their critical importance in post-translational processes, gene silencing and provoking apoptotic cell death in various cancer cell lines (Zhang et al., 2015). Macrophage-derived EVs (Mφ-EVs) are modified using VEGF-siRNA and let-7 miRNA, which target the nucleolin protein (Boimel et al., 2012), nucleolin that plays various roles in the cell, including in the regulation of gene expression, cell proliferation, and tumor development (Burbano De Lara et al., 2021). For this purpose, the DNA aptamer AS1411 (a specific DNA aptamer, a short single-stranded DNA molecule) is used, which has the ability to bind nucleolin with high affinity. The binding of AS1411 to nucleolin interferes nucleolin’s function, and provides inhibition of tumor growth in MDA-MB-231 (Wang et al., 2017; Brenske et al., 2023). CXCL12 have crucial role in breast cancer progression by promoting invasion, angiogenesis and immune system modulation. It can affect the TME by recruiting immunosuppressive macrophages and increasing microvessel density, which supports tumour expansion. Targeting the CXCL12-CXCR4 pathway could be a potential therapeutic strategy for the treatment of breast cancer (Boimel et al., 2012). Another experiment was conducted for the TNBC cell line MDA-MB-231, in which EVs derived from the bone marrow stroma were loaded with several miRNAs, such as miR-127, miR-197, miR-222, and miR-223. These miR targets CXCL12 resulting arrested proliferation in this cell line (Lim et al., 2011). Let-7a miRNA was packed into EVs derived from HEK293 cells and introduced into the TNBC HCC70 cell line. Subsequently, HCC70 cells were successfully implanted into Rag2^−/−^ mice. As a result, it was observed that let-7a miRNA inhibits tumor growth by interfering with the EGFR signaling pathway (Zhou et al., 2014) (Table 2). Previous studies showed that Mesenchymal Stromal/Stem Cell-derived EVs (MSC-EVs) have therapeutic potential for various diseases. Shojaei et al. found that packing miR-381 into MSC-EVs and co-culturing with MDA-MB-231 TNBC cells reduced metastatic capabilities. miR-381 downregulated key transcription factors (Twist and Snail) associated with the Wnt signaling pathway and EMT (Shojaei et al., 2021). NK cells and CD8^+^ T-cells (CTL) are have capacity to detect and kill maligned cells. However, their ability to penetrate deep into tumors is limited. EVs derived from NK cells or CTLs can effectively penetrate solid tumors and help overcome this challenge. TNBC cells often express PD-L1, while its receptor PD-1 is present on tumor-infiltrating lymphocytes (TILs). The interaction between PD-L1 and PD-1 not only attenuates TIL proliferation but also leads to TIL apoptosis, which contributes to the immune evasion mechanism of TNBC. Sthudy shown that PD-1+ EVs released by TILs interact with either the cell surface or PD-L1 of EVs, thereby preventing the interaction between TILs and TNBC cells. As a result, PD-L1-induced suppression of TIL activity is reduced, ultimately increasing the ability of TILs to kill TNBC cells (Das K. et al., 2023) (Table 2).

**TABLE 2:** Extracellular vesicles associated cargos and their roles in TNBC treatment.

S. N.	Extracellular vesicle	Source	Biomolecule	Function	References
1.	Exosomes	THP-1 cells	miR-770	Promoting apoptosis and reduce resistance to chemotherapy	Li et al. (2018)
2.	Extracellular vesicle	Macrophage-derived EVs (Mφ-EVs)	VEGF-siRNA and let-7 miRNA	Inhibit tumor growth in MDA-MB-231cell line	Brenske et al. (2023)
3.	Extracellular vesicle	bone marrow	miR-127, miR-197, miR-222, and miR-223	Arrested proliferation in MDA-MB-231	Lim et al. (2011)
4.	Extracellular vesicle	HEK293 cells	Let-7a miRNA	Inhibits tumor growth by interfering with the EGFR signalling pathway	Zhou et al. (2014)
5.	Extracellular vesicle	Mesenchymal stromal cells (MSCs)	miR-381	Reduced metastatic by downregulated key transcription factors Twist and Snail	Shojaei et al. (2021)

### 3.6 Role of EVs in overcome drug resistance

Engineered EVs as drug carriers offer a promising strategy for overcoming drug resistance in various disease intervention. By facilitating the delivery of therapeutic agents in chemotherapy, targeted therapy, immunotherapy, and endocrine therapy, EV-based approaches offer potential solutions to reduce tumor recurrence and improve clinical outcomes (Zheng et al., 2024). MiR-9 expression is upregulated in temozolomide (TMZ)-resistant glioblastoma (GBM) cells and involves the drug efflux transporter P-gp. Anti-miR-9 loaded EVs is promising strategy for overcoming temozolomide resistance in glioblastoma (Munoz et al., 2013). Thus, engineered EVs highly effective in overcoming various types of drug resistance, such as overcoming reduced drug uptake-induced resistance, for instance, loading cisplatin on EVs derived from M1 and M2 macrophages significantly facilitated drug delivery to resistant neoplasm cells (A2780/DDP) and non-resistant cells (A2780) (Zhang et al., 2020). Similarly, these EVs not only play crucial role in overcoming drug inactivation-induced resistance, overcoming signaling pathway alteration-induced resistance and overcoming apoptosis defect-induced resistance but also, they also can overcome targeted therapy resistance (Zheng et al., 2024). EVs also shown as promising strategy for overcoming mutation-induced resistance, similar to overcoming targeted therapy resistance. For example, imatinib (IM), a selective BCR-ABL inhibitor, mutations in BCR-ABL reduce the binding affinity to IM. Engineered EVs from BCR-ABL siRNA help overcome targeted drug resistance in CML by inhibiting Bcr-Abl (Bellavia et al., 2017). Additionally, various studies have highlighted that overcoming pathway mutation-induced resistance, overcoming immunotherapy resistance, and overcoming endocrine therapy resistance are also effective and promising strategies for the management of TNBC.

### 3.7 EVs in drug delivery

Although most of cells produce EVs, among them not all EVs are suitable for use as drug carriers. EVs that meet quality parameters such as surface protein composition, size, yield, and intracavitary content are considered suitable for drug delivery applications. Various types of cells have been investigated as potential EVs donators for drug delivery such as Dendritic cells (DC), Mesenchymal stem cells (MSC), Macrophages, Milk, red blood cells (RBCs), NK cell-derived EVs, etc. A study demonstrated engineered exosomes (DEVs) could be used to deliver RNA interference (RNAi) therapy into the brain. They used RVG-targeted exosomes, which help deliver treatments directly to brain cells (Meng et al., 2020). MSC-derived EVs (MEVs) exhibit similar functions, including immune modulation, drug delivery, wound healing, and tissue repair, and these features make them promising candidates for therapeutic applications. Increased expression of miR-9 is observed in temozolomide (TMZ)-resistant glioblastoma (GBM) cells (Zhang et al., 2014). EVs have the capacity to deliver targeted drug to specific cells or tissues (Ullah et al., 2021). Additionally, they exhibit the ability to modulate the immune system (Burrello et al., 2016). The utilization of EVs in drug delivery is supported by several advantageous factors. Firstly, EVs exhibit biocompatibility and biodegradability, thereby minimizing potential toxic effects (Chen et al., 2021). In addition, they possess a remarkable ability to selectively target specific cells or tissues through the recognition of surface molecules on vesicles. Additionally, EVs demonstrate proficiency in delivering diverse types of drugs, encompassing small molecules, proteins, and nucleic acids. These attributes render EVs as promising candidates for effective and targeted drug delivery strategies (Peswani Sajnani et al., 2021). EVs for drug delivery show promising potential in diverse therapies. They efficiently carry chemotherapy drugs to tumor cells, thereby enhancing cancer therapy (Meng et al., 2020). In gene therapy, EVs deliver corrective genes that target genetic defects and modulate the immune system to target and destroy oncological cells. In vaccination, EVs provide antigens, inducing a potent and specific immune response, enhancing protection against pathogens (Mehanny et al., 2021).

Researchers have found that EVs have natural properties that help them target specific organs in the body to some extent, and this depends largely on the lipid composition and protein content present on their surface (Murphy et al., 2019). For example, some integrins may alter the pharmacokinetics and cause their accumulation in the brain, lung, or liver, depending on the specific integrin type (Hoshino et al., 2015). Other studies have shown that EVs containing Tspan8 along with integrin alpha4 are more likely to be taken up by pancreatic cells (Rana et al., 2012). In addition, the lipid composition of EVs may also affect the way they are absorbed, as seen with phosphatidylserine, which plays a role in their absorption by macrophages (Matsumoto et al., 2017). Additionally, researchers can further modify the EVs to enhance targeted delivery by engineering producer cells using methods similar to those previously described for cargo loading.

#### 3.7.1 EVs suitable characteristics for drugs delivery

EVs exhibit a lipid bilayer enveloping their entire surface, displaying structural similarity to liposomes (van der Koog et al., 2022). Due to their complex assemblies consisting of lipids, proteins and other bioactive fragments, EVs exhibit exceptional biocompatibility, facilitating pronounced targeting capabilities towards specific cells or tissues (Mathieu et al., 2019). EVs can be bioengineered to contain therapeutic molecules using various technological approaches, including drug incorporation during their biogenesis or post-loading strategies (Song et al., 2022). EVs have intrinsic targeting capabilities due to specific proteins displayed on their surface, which facilitate interaction with target cells or tissues and facilitate precise cargo delivery to the designated site (van Niel et al., 2022). EVs have the natural ability to efficiently and effectively establish themselves in specific cells and tissues within the body (Al-Jipouri et al., 2023). This innate feature enables them to easily cross biological barriers and reach the intended destination easily. As a result, using EVs as carriers for drug delivery greatly simplifies the process, thereby increasing the accuracy and efficacy of drug delivery (Al-Jipouri et al., 2023). As a result, therapeutic agents transported via EVs exhibit enhanced effectiveness and efficiency in their pharmacological actions (van der Meel et al., 2014). EVs exhibit cargo protection capabilities by safeguarding their transported payload from diverse enzymatic and intracellular components, which pose potential risks of cargo degradation (Bahmani and Ullah, 2022). Additionally, the utilization of autologous EVs, which refer to EVs obtained from the patient’s own cells, has the potential to enhance the safety profile of medicines and mitigate the toxicity associated with drug delivery (Elsharkasy et al., 2020). This is attributed to their capacity to reduce immune responses. The utilization of autologous EVs offers a secure and less hazardous approach to drug delivery, particularly for patients susceptible to intense immune reactions or adverse effects (Elsharkasy et al., 2020). Researchers and medical professionals are actively investigating this avenue, as it holds promise for introducing a novel and efficient method of drug administration.

#### 3.7.2 EVs as a delivery vehicle for chemotherapy drugs and biomolecule

Paclitaxel is a chemotherapeutic agent with significant therapeutic implications for various pathology, including breast, lung, and ovarian cancer (Meng et al., 2020). Its primary mode of action involves stabilizing microtubules, which play a critical role in chromosome organization during cell division. Upon binding to microtubules, Paclitaxel impedes their breakdown, resulting in the inhibition of cell division (Honore et al., 2004). Notably, paclitaxel induces apoptosis by activating specific signaling pathways, particularly the p53 pathway, which is responsible for the detection and repair of DNA damage (Ganansia-Leymarie et al., 2003; Kulaberoglu et al., 2021). Additionally, paclitaxel triggers the caspase cascade, which results in DNA fragmentation and degradation of cellular organelles. Despite its efficacy, conventional administration of paclitaxel is associated with unwanted side effects and limited therapeutic benefits (Basu et al., 2020). Encouragingly, preclinical investigations utilizing Paclitaxel-loaded EVs have shown promising outcomes, demonstrating enhanced drug delivery, reduced systemic toxicity, and improved anti-tumor activity (Meng et al., 2020).

A recent study by Xiao Hu and colleagues involved the use of micro-particles to deliver the chemotherapeutic agent paclitaxel (MP-PTX) in combination with radiotherapy for treating TNBC. The experiment demonstrated that targeted delivery of MP-PTX increased absorption, enhancing its ability to kill tumor cells. The combination of MP-PTX and radiotherapy resulted in a synergistic antitumor effect by inhibiting tumor cell proliferation, promoting apoptosis, and reducing the tumor’s immunosuppressive microenvironment. Using MP-PTX and radiotherapy together had a more significant impact on combating TNBC than using them individually(Hu et al., 2023). Another study was conducted by Haney and colleagues, in which they performed experiments using mouse models to treat pulmonary metastasis. They loaded EVs with PTX (EV-PTX) and Doxorubicin (EV-Dox), which effectively targeted malignant cells and showed strong anticancer efficacy in the mouse model of pulmonary metastasis. Subsequently, they developed novel EV-based drug formulations using optimized loading procedures, including variations in pH, temperature, and sonication. These formulations demonstrated high drug loading and efficient accumulation in TNBC cells during *in vitro* testing, indicating a significant anti-proliferation effect of drug-loaded EVs. *In vivo* experiments targeting TNBC in both immune competent and Athymic nu/nu mice resulted in the successful suppression of tumor growth (Haney et al., 2020).

In their research, Gong and colleagues unveiled the presence of A15-Exos, an exosomes containing biomolecules, including disintegrin and metalloproteinase 15, localized on its surface. A15-Exos exhibits the ability to transport DOX and miR-159, thereby targeting TNBC. This combination demonstrates efficacy in treating TNBC by down-regulating the expression of TCF-7 (Gong et al., 2019), a gene promoter involved in cell proliferation, invasion, and metastasis. Moreover, it also suppresses the expression of genes related to cell death, leading to an effective TNBC management without causing adverse effects (Lu et al., 2021). As previously mention modifying the surface of exosomes using several engineering technologies significantly increases drug delivery efficiency, like the CD47-targeted RS17 peptide was encapsulated into liposomes containing the chemotherapeutic agent shikonin, photosensitizer IR820, and immunomodulator poly-metformin for the regimen of BC (Kim et al., 2024). Chlorine E6 (CE6), an FDA-approved photosensitizer, offers a promising approach for targeted pathology treatment in photodynamic therapy (PDT). Integrating Ce6 with 18F-FDG in goat milk-derived exosomes significantly increased the efficacy and accuracy of PDT in BC (Guo et al., 2023). Similarly, TPP-CE6-engineered exosomes were used to load the cancer-specific chemotherapeutic agent pipelongumin (PL). After ultrasonic irradiation, TPP-Ce6-PL-loaded exosomes shows the strongest anticancer effect (Nguyen Cao et al., 2023) (Table 3).

**TABLE 3:** Extracellular Vesicles deliver chemotherapy drugs and biomolecule and its various effect on different target cells.

EVs	Drugs and biomolecule	Target cells	Effect	References
Micro-particles	Paclitaxel (PTX)	MCF-7	PTX increased absorption, enhancing its ability to kill cancer cells. Inhibiting tumor cell proliferation, promoting apoptosis, and reducing the tumour’s immunosuppressive microenvironment	Hu et al. (2023)
A15-Exo	Doxorubicin and miR-159	MDA-MB-231	Down-regulating the expression of TCF-7, resulting cell proliferation, invasion, and metastasis	Gong et al. (2019)
AMSC-Exo	miR-199a		Sensitized cancer cells to the DOX drug and target the m-TOR pathway resulting reduce tumor size, lymph node metastasis.	Wu et al. (2021)
miR-134- EVs	miR-134		Reduced STAT5B and Hsp90, reduced cellular migration and invasion, and enhanced sensitivity to anti-Hsp90 drugs	O’Brien et al. (2015)
HER2+ EVs	Anti-HER2 antibodies and paclitaxel		Reducing side effects and increasing the effectiveness of the treatment	Quinn et al. (2021)
ADSC-exosomes	miR-381	MDA-MB-231 cells	Inhibited proliferation, migration, and invasion by altering EMT-related gene expression	Dong et al. (2022)

In a subsequent investigation Lou and his team isolated exosomes from mesenchymal stem cells obtained from adipose tissues (AMSCs). They modified these exosomes with the biomolecule miR-199a (AMSC-Exo-199a) and administered them to patients with hepatocellular carcinoma. As a result, it was observed that these modified exosomes significantly sensitized disease cells to the DOX drug, thereby targeting the mTOR pathway (Lou et al., 2020). The mTOR pathway is linked to various poor prognostic factors, including increased tumor size, lymphnode metastasis, and shorter survival (Wu et al., 2021) (Table 3).

## 4 Limitation of extracellular vesicle in TNBC

The diversity of EVs and their presence in various types of diseases has captivated the scientific community. However, their application is currently constrained by issues related to cargo delivery, biological barriers, and clinical translation. Researchers have been actively engaged in addressing these limitations of EVs and are diligently working towards harnessing their substantial potential as a powerful tool in the management of TNBC. One important reason for the failure of TNBC management is the lack of a specific molecular target that can be focused on (Maqbool et al., 2022). On the other hand, EVs have been explored as a crucial biomarker and prognostic tool for TNBC (Dong et al., 2022). However, recognizing a specific target of TNBC is currently a challenge in order to deliver therapeutic cargo through EVs (Haney et al., 2020). Additional off targeting a major challenge, because EVs membranes are enriched with receptors or ligands that interact with target cells, providing them with inherent targeting missions. But most natural therapeutic EVs suffer the fate of being cleared by macrophages, resulting EVs often become off target and fail to reach their destination. Therefore, it is necessary to reduce this off-target effect to improve the bioavailability of target tissues. Despite EV-based drug delivery is now a breakthrough for clinical pathology. Methods such as incubation provide a simple and non-disruptive approach to loading drugs into EVs. However, limited drug-loading capacity remains a significant drawback (Gangadaran and Ahn, 2020).

The low toxicity, immune tolerance and long half-life of EVs has been a boon in the medical field. However, their slow secretion remains a major obstacle to clinical applications. Over the past decades, there has been an emphasis on large-scale EV production using physical, chemical, and environmental stimulation methods. However, none of these approaches have been successful enough to be widely adopted for clinical applications (Hahm et al., 2021). Among the many challenges, some of these like Complexities in isolating EVs from various biological mixtures arise from their inherent heterogeneity in their size, shape and composition (Ding et al., 2021). This challenge is further compounded by variation in EVs secretion rates from biological source and different cell types, which can reduce yields and limit sample size, hindering thorough analysis (Dong et al., 2022). Maintaining the functional behavior of EVs remains a major challenge. EVs obtained from MSCs or other parts of the body remain stable and viable for long periods at −80°C, but freezing and thawing can lead to clustering. Storage and transportation at low temperatures may reduce the translational activity of EVs. This drawback can be overcome by using freeze-dried exosomes, that allows storage at room temperature, extends shelf life, and reduces storage and transportation costs (Karn et al., 2021).

Additionally, the presence of interfering biological molecules like proteins, lipids, and nucleic acids complicates the precise isolation and purification of EVs (Mateescu et al., 2017). The lack of a standardized method to isolate EVs from various bodily sources also presents a challenge (Zeng et al., 2022). Currently, five EV isolation methods include ultracentrifugation, size-based techniques, immunoaffinity capture, precipitation, and microfluidics (Yang et al., 2020). Ultracentrifugation is most widely used method, with other methods being used by only 5%–20% of researchers (Li and Xu, 2019). Each technique has advantages and disadvantages. Differential ultracentrifugation is cost-effective but inefficient and potentially harmful to EVs during recovery (Tiwari et al., 2021). On the other hand, Density gradient centrifugation is more efficient method for recovering EVs, but it is also more expensive and time-consuming (Monguió-Tortajada et al., 2019). The Immunoaffinity method is effective but time-consuming and costly. Similar challenges exist with other methods (Taylor and Shah, 2015). Given these limitations, there is a growing demand for user-friendly, affordable tools that efficiently isolate EVs while saving time (Yuan et al., 2021).

The entrapment of diverse cargo within EVs presents a formidable challenge, stemming from the geometrical constraints imposed by the small dimensions of EVs that reduce their viability for large molecular species (Tran and Tran, 2020). The phospholipid bilayer constitution of EV membranes imposes a selective barrier against macromolecules with high electrostatic potential, and concurrently selective cargo units are endowed with surface-bound receptors on EVs, which makes their preferential interaction possible (van der Meel et al., 2014). Nevertheless, the lack of receptors in all cargo categories prevents binding interactions, thereby hindering the association. ATP, apart from its role as a universal energy currency, assumes pivotal significance in the context of encapsulating bio-macromolecules within EVs, harnessing energy sourced from ATP hydrolysis to facilitate and potentiate cargo encapsulation processes. The depletion of ATP stores can complicate this complex process (Russell et al., 2019).

EVs offer a promising avenue for disease diagnosis, but this promise is accompanied by certain challenges. Variability in samples, whether derived from blood, urine, saliva or other sources, can result in variation in the amount and type of EVs (Jafari et al., 2020). Research focusing on EVs biomarkers often adopts diverse criteria to indicate specific EVs subpopulations, potentially leading to inconsistent findings across different studies. To mitigate this problem, standardization of these criteria becomes imperative to establish reliable and consistent diagnostic markers (Yan et al., 2021). Moreover, the sensitivity of diagnostic tests based on EVs can be influenced by multiple factors including the selection of detection methods (such as ELISA, flow cytometry, and nanoparticle tracking analysis) and the quality of reagents employed (Serrano-Pertierra et al., 2020). Considering that EVs exhibit heterogeneity in both size and composition, the presence of analogous-sized cellular fragments or vesicles might impede the precise detection of targeted biomarkers, underscoring the need for methodological refinement in this promising diagnostic approach.

Our understanding of EVs’ role in supporting TNBC, their mechanisms, and their impact on TNBC interventions is currently limited (St-Denis-Bissonnette et al., 2022). The assistance of EVs in TNBC metastasis and their role in epigenetic processes like methylation, histone modification, and non-coding RNA activities remains unclear (Zolota et al., 2021) Additionally, roles and alterations in immune regulation, forming pre-metastatic niches in metastatic organs, lack comprehensive understanding and require further research (Yang et al., 2021). The complex BC tumor microenvironment, with diverse cells and interactions, poses challenges in understanding TNBC progression’s regulatory mechanisms. Uncertainties persist about specific cell-derived EV components crucial in this network (Awadasseid et al., 2021). Many research studies concentrate solely on specific targets within EVs, preventing a comprehensive perspective. It's crucial to precisely track the path, dispersion, and destiny of EVs to comprehend how they are absorbed and their impacts on the recipient cells. The complex structure of exosomes and their interactions with recipient cells remain mysterious, underscoring the insufficient understanding of the mechanisms through which EVs operate in TNBC. Bridging these knowledge gaps plays an important role in realizing the potential of EVs as a diagnostic aid and therapeutic focal point in dealing with TNBC.

## 5 Future directions of EVs application as therapeutics in TNBC

The evidence highlighting the role of EVs in TNBC has presented another opportunity for research, particularly in the development of EVs as diagnostic/prognostic biomarkers, but more importantly, as therapeutic agents. EVs demonstrated promising results in cancer vaccination and drug delivery, making it a noteworthy consideration in medical applications. The initial characterization of EVs reveals their unique therapeutic properties, making them interesting for potential use in clinical field (Elsharkasy et al., 2020). EVs encapsulate nucleic acids and proteins released from malignant cells, providing valuable insights into the essential characteristics of disease cells (Cerezo-Magaña et al., 2020). Studies results shown that EVs can indicate differences between tumor samples and controls, which make it easier to obtain preliminary information about disease prognosis (Hoshino et al., 2020). The ability to detect such differences has the potential to revolutionize the approach to BC diagnoses. Through the examination of the contents of EVs, medical professionals could enhance their comprehension of the ailment and its advancement, ultimately resulting in improved and individually tailored therapies.

## 6 Conclusion

TNBC shows significant challenges in its management due to its aggressive nature, propensity for metastasis, and limited strategy options. Current therapies, including surgery, chemotherapy, and radiation, have shown limited efficacy, particularly in addressing metastatic tumors. Limitations of currently used diagnostic tools like imaging (mammography, ultrasound, and magnetic resonance imaging) and immunohistochemistry (IHC) in the diagnosis of TNBC, make challenging to obtain comprehensive and accurate information about it. EVs opened new avenues for the diagnosis of various disease including TNBC. EVs associated protein biomarkers such as EGFR, CCL5, CD24, ADAM10, cofilin-1 and Non-coding RNA (ncRNA) like miRNA have significant role in its diagnosis as previous mention. In the field of oncology, the integration of AI, especially DL, significantly enhances tumor diagnosis, intervention, and prognosis. With its ability to automatically extract features and analyze large datasets, DL has revolutionized medical imaging, improving diagnostic accuracy while reducing false positives. The inability to effectively manage TNBC so far is largely due to drug resistance, which remains a major challenge. This challenge can overcome by EV-associated biomolecules, and EVs are capable of carrying molecules, and allowing non-invasive monitoring of the disease. The emergence of EVs as versatile mediators of intercellular communication and potential therapeutic carriers offers a promising avenue for the development of more effective TNBC therapeutic approaches. EVs also have crucial role in the development of TNBC and significantly influence patient outcomes. EVs possess inherent properties that make them well-suited for targeted drug delivery, ability to evade the immune system, and capacity to traverse biological barriers like the blood-brain barrier. The use of EVs as drug delivery vehicles has the potential to enhance intervention accuracy, efficacy, and patient outcomes in TNBC and other malignant diseases, representing an attractive area for future research and clinical exploration.
